# Autopsy of all young sudden death cases is important to increase survival in family members left behind

**DOI:** 10.1093/europace/euae128

**Published:** 2024-05-08

**Authors:** Thomas H Lynge, Christine M Albert, Cristina Basso, Rodrigue Garcia, Andrew D Krahn, Christopher Semsarian, Mary N Sheppard, Elijah R Behr, Jacob Tfelt-Hansen

**Affiliations:** The Department of Cardiology, The Heart Centre, Copenhagen University Hospital, Rigshospitalet, Copenhagen, Denmark; Department of Cardiology, Smidt Heart Institute, Cedars-Sinai Medical Center, Los Angeles, CA, USA; The Cardiovascular Pathology Unit, Azienda Ospedaliera, Department of Cardiac, Thoracic, and Vascular Sciences and Public Health, University of Padua, Via Aristide Gabelli, 61, 35121 Padova PD, Italy; Department of Cardiology, Poitiers University Hospital, Poitiers, France; Center for Cardiovascular Innovation, Heart Rhythm Services, Division of Cardiology, University of British Columbia, Vancouver, British Columbia, Canada; Agnes Ginges Centre for Molecular Cardiology, Centenary Institute, The University of Sydney, Sydney, Australia; Cardiovascular Pathology Unit, Cardiovascular and Genetics Research Institute, St George’s, University of London, St George’s University Hospitals NHS Foundation Trust, London, UK; Cardiovascular Clinical Academic Group, Cardiovascular and Genetics Research Institute, St George’s University of London and St George’s University Hospitals NHS Foundation Trust, London, UK; The Department of Cardiology, The Heart Centre, Copenhagen University Hospital, Rigshospitalet, Copenhagen, Denmark; The Department of Forensic Medicine, Copenhagen University Hospital, Rigshospitalet, Copenhagen, Denmark

**Keywords:** Sudden death, Sudden cardiac death, Autopsy, Prevention, Inherited heart disease, Cause of death

## Abstract

Sudden cardiac death (SCD) is an important public health problem worldwide, accounting for an estimated 6–20% of total mortality. A significant proportion of SCD is caused by inherited heart disease, especially among the young. An autopsy is crucial to establish a diagnosis of inherited heart disease, allowing for subsequent identification of family members who require cardiac evaluation. Autopsy of cases of unexplained sudden death in the young is recommended by both the European Society of Cardiology and the American Heart Association. Overall autopsy rates, however, have been declining in many countries across the globe, and there is a lack of skilled trained pathologists able to carry out full autopsies. Recent studies show that not all cases of sudden death in the young are autopsied, likely due to financial, administrative, and organizational limitations as well as awareness among police, legal authorities, and physicians. Consequently, diagnoses of inherited heart disease are likely missed, along with the opportunity for treatment and prevention among surviving relatives. This article reviews the evidence for the role of autopsy in sudden death, how the cardiologist should interpret the autopsy-record, and how this can be integrated and implemented in clinical practice. Finally, we identify areas for future research along with potential for healthcare reform aimed at increasing autopsy awareness and ultimately reducing mortality from SCD.

## Graphical Abstract

**Graphical Abstract:**
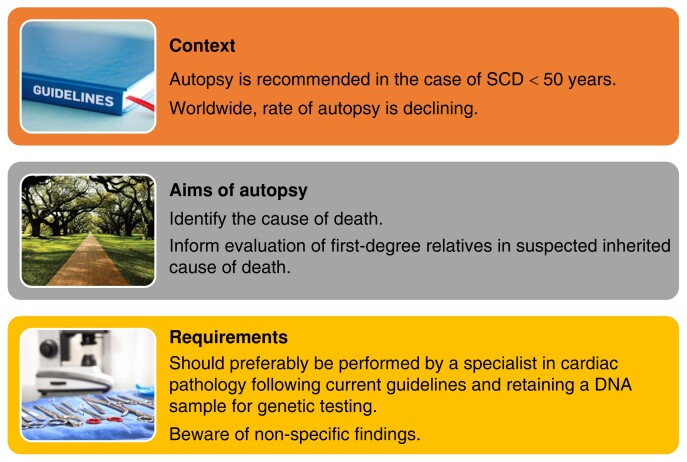
Autopsy in sudden cardiac death

## Introduction

Sudden cardiac death (SCD) is a major public health problem worldwide and an important cause of both cardiovascular and total mortality.^1–11^ A significant proportion of SCD occurs in people of working age, and SCD consequently constitutes a significant societal burden in addition to the immense personal consequences of these events.^7,12–18^

Sudden cardiac death is inherently difficult to prevent due to the suddenness and often unexpected nature of the event. Previous studies suggest that approximately 50% of cases occur in individuals not previously diagnosed with cardiovascular disease and often as the first manifestation of disease.^1,16,19–21^ Although there have been advances in cardiopulmonary resuscitation and post-resuscitation care resulting in increased survival following cardiac arrest, the survival rates remain <10% in most countries.^4,8,16,20,22–26^ It follows that primary prevention of sudden cardiac arrest is essential to reduce SCD mortality.

It has previously been shown that the majority of SCD in young persons aged 1–35 years is caused by potentially inherited cardiac diseases, including inherited structural cardiac disease along with primary arrhythmogenic disorders.^13–15,27–32^ Furthermore, recent studies indicate that inherited cardiac diseases continue to underlie a significant proportion of SCD among cases aged 36–49 years, whereas the prevalence of inherited cardiac disease in SCD cases > 50 years is poorly understood.^1,4,7,8,12,15–17,19,27,28,33–37^

Identification of inheritable causes of SCD provides a means to lower the risk of SCD in family members. Autopsy and post-mortem genetic testing of young SCD cases along with detailed cardiac and genetic investigations of first-degree relatives result in high yield of diagnoses of inherited cardiac disease in the families.^13,17,38–50^

The aim of this review with a joint effort from experts in arrhythmias and genetic heart disease as well as cardiac pathology is to examine the evidence of the utility of autopsy in sudden death (SD) in the young. Next, we provide an expert consensus statement on how autopsy can be integrated and implemented in clinical practice. Finally, we identify areas for future research aimed at reducing the incidence of SCD in the general population.

## Methods

A PubMed literature search was conducted with focus on articles published within the last 20 years, with the latest search taking place on 11 January 2023. The search was conducted using various combinations of the following terms: sudden cardiac death, sudden death, and autopsy. All papers written in English with information on autopsy in SD cases were included. The eligibility of all papers was determined by review of both abstract and full text. All papers referenced in the reviewed articles were also evaluated for relevant content. Finally, Web of Science was used to identify all articles citing the reviewed articles, and these were scrutinized for relevance.

## Results and discussion

### Definitions and epidemiology

The terms used in this review are defined in *Table 1*. Definitions of SD and SCD have varied between previous studies, and there has been disagreement between what constitutes both ‘sudden’ and ‘unexpected’. Most recent studies use a SD definition with a time constraint and distinguish between witnessed and unwitnessed cases, where witnessed cases have to occur within 1 h of change in cardiovascular status and unwitnessed cases have to be seen alive and functioning normally within 24 h of being found dead.^7,12–15,28,51–58^

**Table 1:** Definitions of common terms used in this review

Term	Definition
Sudden cardiac arrest (SCA)	Sudden cessation of normal cardiac activity with haemodynamic collapse
Sudden cardiac death (SCD)	Sudden natural death of presumed cardiac cause that occurs within 1 h of symptom onset in witnessed cases and within 24 h of last being seen alive in unwitnessed cases. Sudden cardiac death in autopsied cases is defined as the natural unexpected death of unknown or cardiac cause.
Sudden unexplained/unexpected death	Sudden death occurring in a person > 1 year old in which previous medical history does not give a cause
Sudden arrhythmic death syndrome (SADS)	Unexplained sudden death occurring in a person > 1 year old with negative pathological and toxicological assessment
Sudden infant death syndrome (SIDS)	Unexplained sudden death occurring in a person < 1 year old with negative pathological and toxicological assessment and circumstantial and forensic evaluation

The authors of a recent paper by researchers from the European Sudden Cardiac Arrest network—towards Prevention, Education, and New Effective Treatments (ESCAPE-NET) consortium have suggested a practical approach to identification of SCD cases using a stratified approach dividing the SCD cases into definite, possible, and probable SCD to reflect the level of certainty of diagnosis and degree of information.^57^ Many of the authors of the present review co-authored this publication, and we fully support this approach for SCD identification in a research setting.

**Figure 1:**
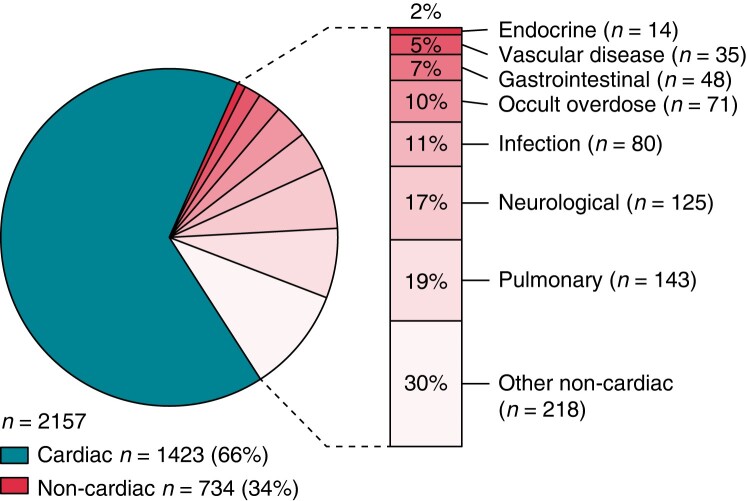
Autopsy-defined causes of presumed SCD. Composite data from three population-based studies: cardiac deaths *n* = 1423 (*n* = 315, Tseng *et al.*^53^; *n* = 355, Haukilahti *et al*.^63^; *n* = 753, Risgaard *et al*.^59^); non-cardiac deaths *n* = 734 (*n* = 210, Tseng *et al*.^53^; *n* = 238, Haukilahti *et al.*^63^; *n* = 286 Risgaard *et al.*^59^). Reprinted from Marijon *et al.*^9^.

The majority of SD has a cardiac cause, i.e. SCD, but many may also be due to non-cardiac causes such as cerebral haemorrhage, pulmonary embolism, asthma, and drug toxicity (*Figure 1*).^3–5,16,59–62^ Multiple studies have attempted to estimate SCD burden in the general population, and although most agree that SCD burden is significant, the reported annual SCD rates range widely from 15 to 159 per 100 000 persons, which corresponds to 6–20% of all deaths.^1,4,7,8,12,16,19,27,28,33–36^ The high degree of variability in these results are in part due to differences in definitions of SCD, case ascertainment criteria, and methods for estimation of incidence.

**Figure 2:**
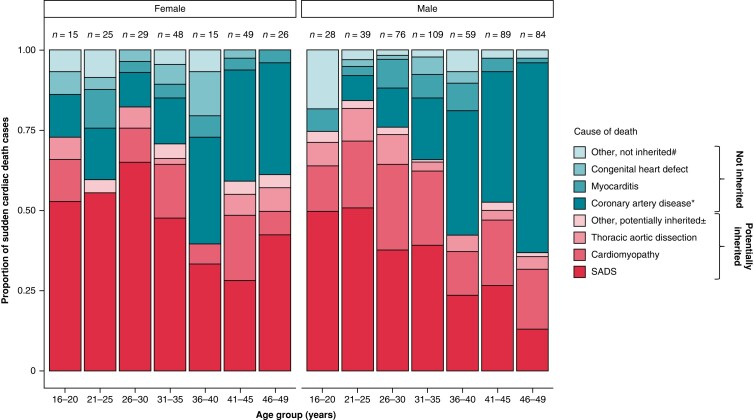
Sex-stratified distribution of causes of death among autopsied cases of sudden cardiac death according to age in persons aged 16–49 years in Denmark. SADS, sudden arrhythmic death syndrome. Reprinted from Lynge *et al.*^17^.

The incidence and causes of SCD are highly dependent on age and sex (*Figure 2*).^17,64,65^ The lowest annual SCD incidence is reported in children, adolescents, and young adults, while infants (<1 year) have a higher SD incidence.^9–12,23–26^ From the age of 35 years, SCD incidence increases markedly up until the age of 60–80 years. The relatively high SD incidence in infants is mainly driven by deaths caused by sudden infant death syndrome (SIDS) and congenital heart defects.^66–68^ Previously, a ‘triple risk’-hypothesis has been proposed suggesting that SIDS results from a convergence of three overlapping risk factors: a vulnerable infant, a critical developmental period, and an exogenous stressor.^69^ Underlying vulnerability includes both non-genetic (e.g. *in utero* exposure to smoking and alcohol and poor foetal growth) and genetic factors.^70^ Although most SIDS cases seem to have a non-cardiac background, growing evidence point towards a subset of cases caused by infantile presentations of inherited structural heart disease and primary arrhythmia such as Long QT Syndrome.^70–77^ Previous studies suggest that up to 14% of SIDS cases have an ultra-rare gene variant related to inherited cardiac conditions that might have caused an arrhythmic SD of the infant.^70^

The majority of SCD in young persons aged 1–35 years is caused by potentially inherited heart disease such as arrhythmogenic cardiomyopathy, hypertrophic cardiomyopathy, and dilated cardiomyopathy along with primary arrhythmogenic disorders, including Brugada syndrome, catecholaminergic polymorphic ventricular tachycardia (CPVT), and congenital long QT syndrome.^13–15,27–30,37,78–82^ The steep increase in SCD incidence from the age of 35 years is largely driven by ischaemic heart disease, which is also heritable to some degree and especially in young cases can result from monogenic inherited disease such as familial hypercholesterolaemia. Potentially inherited non-coronary heart disease, however, remains a common cause of death in SCD cases up until the age of 50 years.^1,4,7,8,12,15–17,19,27,28,33–36,83^ There is limited information on causes of SCD in persons above the age of 50 years, although it is generally accepted that ischaemic heart disease is the most common cardiac pathology underlying SCD in this age group.^4,16,19,84,85^

Across all ages, males have higher risk of SCD compared with females, even after adjustment for common risk factors of ischaemic heart disease.^16,86^ Ethnic background has also been shown to influence the risk of SCD.^16,87,88^ Notably, studies from the USA have shown higher rates of SCD among people with African origin, while estimates of SCD burden in Asian countries are consistently lower when compared with Western European countries and the USA.^4,16,89,90^

### Autopsy in sudden death

Autopsy is the gold standard for identifying cause of death, and in most cases, categorization of a definite SCD requires either an autopsy, or an observed primary ventricular arrhythmia immediately before death.^1,91^ Sudden cardiac death is often the first manifestation of disease and autopsy in these cases is the only opportunity to establish and register an accurate cause of death. Previous studies show that regardless of whether death occurs inside or outside of the hospital, the cause of death as stated on the death certificate is incorrect in a significant proportion of autopsied deaths.^92,93^ In a US autopsy study, the overall sensitivity of the death certificate in predicting an individual cause of death was 0.47 with even lower sensitivity (0.28) in predicting death from cardiovascular causes.^92^

In a recent study by Tseng *et al*.^53^ of 525 presumed SCD cases, 40% were shown to have either unnatural or non-cardiac causes after autopsy. Furthermore, results from a Danish study showed a non-cardiac cause of death in 28% of all autopsied SD cases among the young.^59^ These results highlight the key role of the autopsy in combination with toxicology in correctly identifying SCD cases and diagnose the underlying cause. The interpretation of the autopsy findings, however, should always be carried out with great care and viewed in the context of the clinical circumstances. In the above-mentioned studies, some cases of primary electrical disorders could potentially be missed due to the risk of erroneously attributing cause of death to a non-specific structural non-cardiac finding.

**Figure 3:**
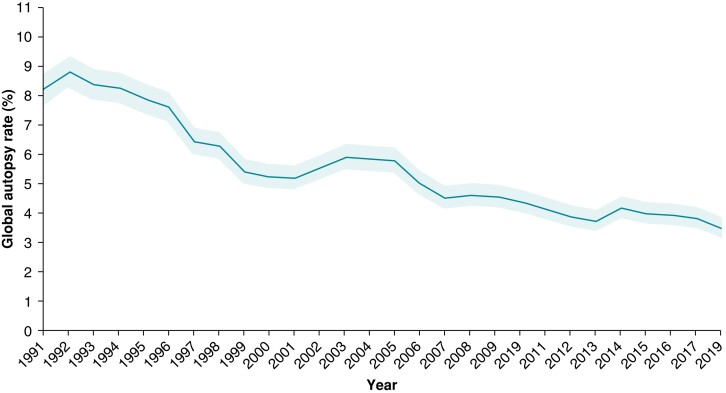
Annual global autopsy rate (95% CI) of all deaths in west European countries from 1991 to 2018 according to the World Health Organization. The annual autopsy rate for all deaths in Austria, Finland, the Netherlands, Portugal, Switzerland, Denmark, Iceland, Luxembourg, Norway, Sweden, and the UK from 1991 to 2018 without distinction between clinical or medicolegal autopsy. Reprinted from Marijon *et al.*^9^.

Over recent decades, however, there has been a decrease in autopsy rates in many countries across the globe (*Figure 3*).^47,91,94^ In general, increasing age and comorbidity burden are associated with lower rates of post-mortem examination, including both medicolegal external examination and autopsy.^15,22,47,95,96^ This is problematic as it is well known that several comorbidities such as diabetes mellitus, kidney disease, and epilepsy are associated with higher risk of SD.^97–99^ Autopsy is not always performed in cases of SD, even in those below the age of 50 years.^1,17,47^ In a recent study of autopsy practice across several European countries conducted on behalf of the Association for European Cardiovascular Pathology, autopsy was not performed in up to 40% of SD cases < 50 years old.^100^ This was primarily due to monetary reasons or lack of interest among police, legal authorities, and physicians. In addition, only 50% of pathologist followed a standard protocol for autopsy, apparently due to lack of expertise and/or training. However, in the UK, all sudden unexpected deaths without a natural explanation are mandated to have an autopsy done by a fully trained pathologist, which has maintained the autopsy rate at approximately 100 000 autopsies per annum, permitting establishment of a large UK SCD database derived from referrals of several hundred autopsied SCD cases for expert cardiac pathological examination per year.^31^

Autopsy in SD cases has several purposes: (i) identification of unnatural causes of death, including drug abuse and examination of potential criminal or third person involvement; (ii) identification of natural causes of death, specifically distinguishing between non-cardiac and cardiac causes; and (iii) identification of potentially inherited causes of SCD. Often coroners or medical examiners are only required to address the first purpose.

There are large variations in the conduct of autopsies between countries. These are performed as either clinical or forensic autopsies.^47^ Clinical autopsies are conducted at local hospital pathology departments at the request of the deceased patient’s treating physician or general practitioner and, in some countries, next of kin. These autopsies include macroscopic and histological examination, while additional examination such as genetic evaluation, toxicology, and microbiological examination are not routinely performed.

**Figure 4:**
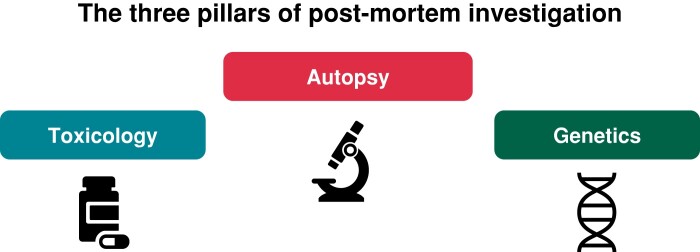
The three pillars of post-mortem investigation.

Forensic autopsies on the other hand are performed if the cause of death has not been established with certainty, and the death is an unexpected and unexplained SD, especially among the young.^17,47^ Again, there is significant variation according to national law, but these autopsies may be requested by a coroner, magistrate, public prosecutor, official death investigator, or the police to assist in determining cause and manner of death. Ideally, all autopsies follow a standardized protocol, in which all organs are examined (*Figure 4*).^47,86,101^ The UK Royal College of Pathologists, Society of Cardiovascular Pathology, and the Association for European Cardiovascular Pathology have published recommended guidelines for autopsy investigation of SCD cases.^47,102,103^ The Association for European Cardiovascular Pathology has also recently issued guidelines for diagnosis of genetic cardiomyopathies at autopsy.^48^ Blood or spleen tissue should routinely be saved for potential toxicological and genetic testing if deemed necessary, and the examinations may also include biochemistry and microbiology in selected cases.^56^

We know from previous studies that expert cardiac pathology evaluation is essential as this significantly improves the post-mortem diagnostic accuracy.^100,104^ For example, an English study from 2014 found that the initial diagnosis of cause of SCD made by the referring pathologist was altered after expert evaluation in 41% of cases.^104^ For Arrhythmogenic Right Ventricular Cardiomyopathy, only 2 out of 20 were confirmed by the expert cardiac pathologist highlighting the difficult task of cardiac pathology postmortem.^104^

### Yield of post-mortem investigation

In order to correctly diagnose causes of SD, autopsy together with premorbid clinical information is essential.^86,100,105^ It is, therefore, pivotal in cases of SD to collect all available information on family history, prior symptoms, and diseases of the decedent, along with circumstances of death. If suspicion of an inherited cause of SCD remain after initial evaluation, including an autopsy without a non-cardiac cause or an acquired non-inherited cardiac cause, it is important to secure blood/tissue for potential genetic testing.^46,47,106^ It is also vital to perform toxicological testing to exclude drug overdose as these cases do not warrant further cardiac investigation.^53^

In young SCD cases (<50 years old), a potential genetic disease can be identified in up to 50% of cases.^17,38,86,107^ Conventional autopsy will identify likely heritable disorders that can cause structural cardiac disease, such as cardiomyopathies, premature ischaemic heart disease, and aortopathies. In around 40–60% of autopsied cases of SCD in the young, the cause of death remains unexplained after autopsy and toxicological examination.^13–15,31,37,108^ These deaths are often caused by primary arrhythmogenic disorders such as long QT syndrome, Brugada syndrome, and CPVT and have been termed sudden arrhythmic death syndrome (SADS) to highlight this potential aetiology. Recently, a novel phenomenon termed concealed cardiomyopathy has emerged as studies have shown that there is also a yield of pathogenic variants in cardiomyopathy genes in structural normal heart.^38^ A recent study showed that the yield of cardiomyopathy genes seems to be higher in SADS cases, where the autopsy was inconclusive.^109^ Other non-cardiac causes may underlie these deaths, such as epilepsy, although more research is still currently required to determine their significance in SADS.^98,110,111^

If autopsy identifies a potentially inherited structural heart disease, targeted disease-specific genetic testing should be performed according to current guidelines, and first-degree relatives should be referred for cardiac assessment.^86,112^ A case series of individuals who have been diagnosed with cardiomyopathy at autopsy has indicated that around one-third harbour pathogenic and likely pathogenic variants in the genes responsible for these disorders.^113^

Post-mortem genetic testing in SADS cases, also termed the molecular autopsy, initially focused on sequencing the four main genes commonly associated with long QT syndrome, Brugada syndrome, and CPVT (SCN5A, KCNQ1, KCNH2, and RYR2) with yields of potentially actionable genetic variants of around a quarter.^114^ Thus, consensus statements have focused on testing for primary electrical diseases if the decedent is young (<50 years) and/or the circumstances of death and/or the family history support a primary electrical disease.^86^

Contemporary studies have, however, shown lower yields of pathogenic and likely pathogenic variants.^115^ More recent research has employed large panels ranging from 50 to 250 genes implicated in cardiac disease.^106,116–118^ In a large prospective study of 490 SCDs in children and young adults in Australia and New Zealand, Bagnall *et al*.^13^ found that a potentially clinically relevant cardiac gene variant was identified in 27% of unexplained SCD cases, just over 100 of whom had DNA for which genetic testing was performed. In a later study from 2017, post-mortem genetic testing in over 300 cases of SADS resulted in a yield of 13% for pathogenic and likely pathogenic variants in cardiac-related genes.^38^ If genetic evaluation was combined with thorough cardiac evaluation of relatives, the diagnostic yield of genetic cardiac diseases was as high as 39%.^38^ However, the additional diagnostic genetic variants found may indicate that cardiomyopathy was the likely cause of death in some cases, despite the negative autopsy, suggesting that concealed cardiomyopathy may be present in the decedent.^38,109^

In recent years, there have been a promising progress in genetic discoveries, which integrated with detailed clinical data will allow for a more comprehensive genetic evaluation in SADS families.^13,38,44,56,86,119^ For example, recent research has highlighted that calmodulinopathies caused by mutations in one of three genes that encode identical calmodulin protein (CALM 1–3) can cause life-threatening arrhythmias and SCD.^120,121^

We recommend the use of a broad ‘molecular autopsy’ panel of genes that have been robustly linked to primary arrhythmia syndromes and cardiomyopathies such as that recommended in the UK National Health Service and adjudicated through PanelAPP.^113^

### How should the cardiologist read the autopsy report?

The quality and detail of the autopsy report are vital to establish a cause of SD but vary widely across pathology and forensic units, both within countries and across the world. The cardiologist leading the multidisciplinary team should read through the autopsy report in detail and be wary of subtle findings which are ‘non-specific’ and therefore not diagnostic but may be of clinical significance, such as focal myocardial fibrosis, fibro-fatty tissue replacement, and mild cardiomegaly, which may represent early cardiomyopathic changes. The corollary is also true, where abnormalities likely caused by prolonged resuscitation confound the attributed cause of death, leading to misinformation.^122^ Furthermore, some non-specific findings such as isolated fatty infiltration or minimal inflammatory infiltrates may represent bystander phenomena irrelevant to the cause of death.^123^ In the setting of these non-specific findings, genetic testing will add to the diagnostic accuracy. Toxicology findings need to also be noted, along with the circumstances of the death. The cardiologist’s review of the autopsy, the interaction with the pathology/forensic physicians who performed the autopsy, and the request for a second opinion from an expert cardiovascular pathologist when the autopsy report is unclear may shed light on the potential cause of SD, with important implications for the family.

### Follow-up and management of family members of sudden cardiac death victims

**Figure 5:**
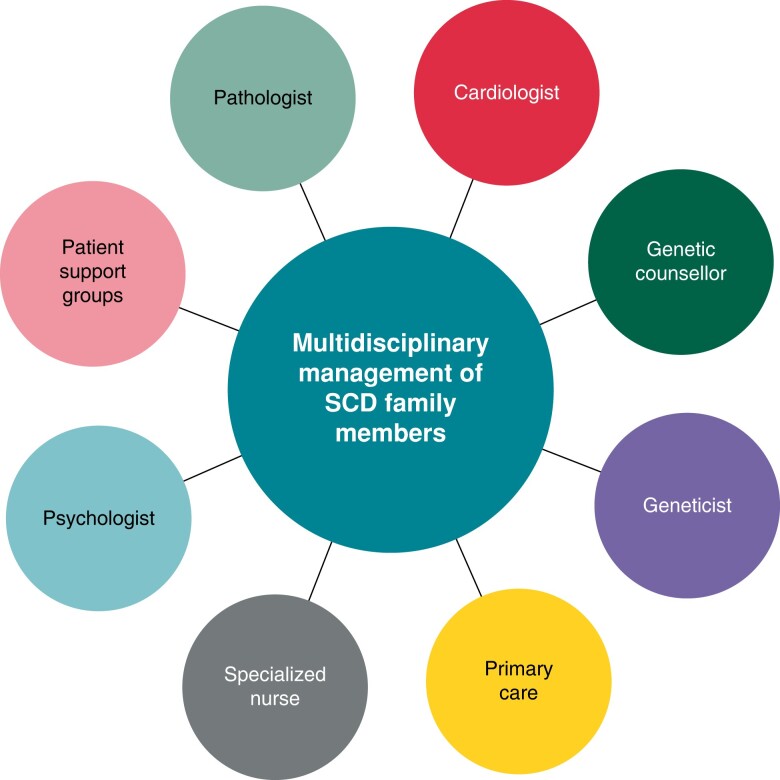
Overview of a multidisciplinary approach to the management of families of sudden cardiac death cases.

Both the clinical and genetic diagnoses and subsequent follow-up of family members of SCD cases with a suspected inherited basis are complex and require expertise in both genetic aspects of the disease as well as clinical cardiovascular investigation and management and care for the psychosocial well-being of the families.^22,48,56,117,123,124^ In line with previous consensus statements and guidelines, we recommend the use of a multidisciplinary approach by pathologists, cardiologists, genetic counsellors, geneticists, specialized nurses, clinical psychologists, and patient support groups (*Figure 5*).^46,47,56,117^ In Denmark in 2006, specialized hospital-based units with a particular focus on inherited cardiac disease were implemented in recognition of the need for a dedicated and specialized effort with close cross-sectional collaboration. To our knowledge, there are no studies providing direct evidence of an effect of such specialized hospital units. The incidence of SCD among the young in Denmark has, however, been declining, although this is likely to be multifactorial, including improved treatment of out-of-hospital cardiac arrest.^57^

Follow-up is based on the thorough premorbid clinical and post-mortem examination of the decedent. In case of a non-autopsied SCD in a young person where circumstances or family history suggest an inherited cardiac disease, we recommend that first-degree relatives are referred for familial evaluation in a specialized clinic.^86^ The subsequent examination and assessment, however, are complicated by the inability to exclude that the death could have been caused by a non-inherited disease, including drug overdose, non-inherited cardiac disease (e.g. myocarditis), and non-cardiac disease.^53,59^ This again highlights the importance of autopsy in all cases of SD in the young, as identification of a non-inheritable cause of death prevents unnecessary and potentially harmful examinations and treatments, and spares the family the worry of increased risk of SD.^125^

Ideally, the first family members to be assessed are the parents of the deceased. Overall, detailed cardiac and genetic investigations of first-degree relatives of SADS cases or SCD on the basis of potentially inherited structural heart disease result in a diagnosis of inherited cardiac disease in 20–50% of the families,^42–44,113,123,126,127^ although the yield depends on the protocol employed.

If autopsy identifies a potentially inherited structural heart disease, it is recommended to refer first-degree relatives, obligate carriers, and relatives with relevant symptoms to a specialized multidisciplinary clinic for cardiac assessment and targeted genetic testing.^86,127,128^ The latter is influenced by performance and applicability of genetic testing in the decedent.

**Figure 6:**
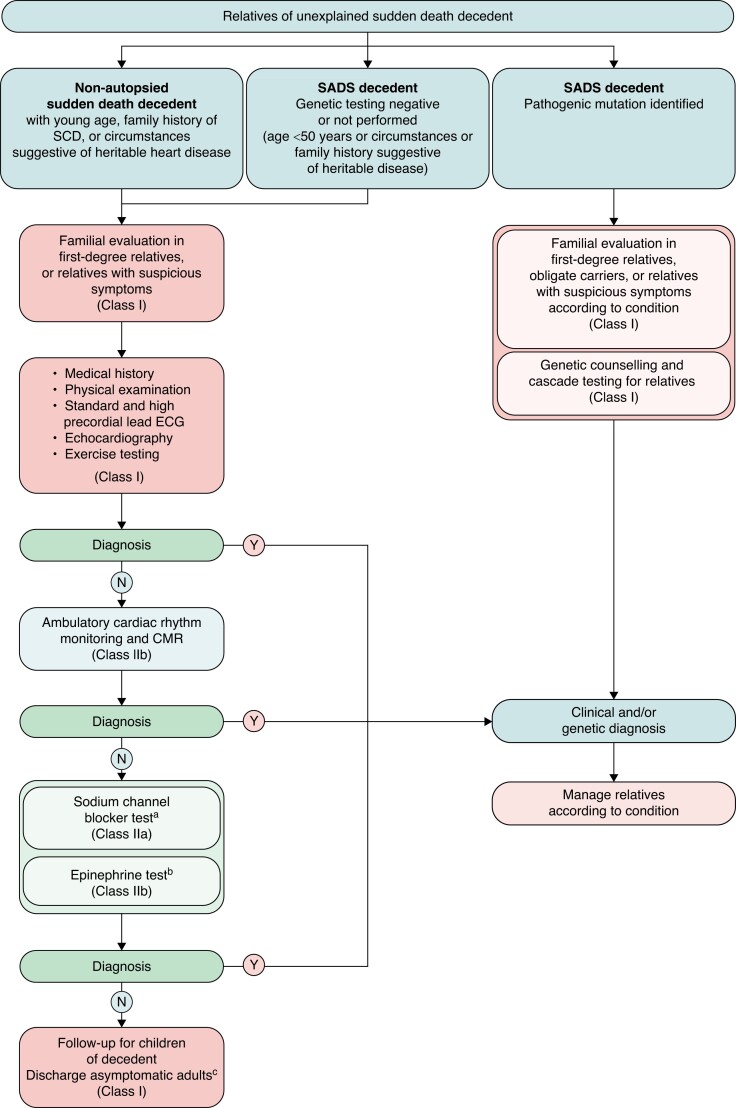
Algorithm for the evaluation of relatives of unexplained sudden death decedents. CMR, cardiac magnetic resonance; SADS, sudden arrhythmic death syndrome; SCD, sudden cardiac death. ^a^>16 years old + any suspicions for Brugada syndrome on tests or decedent circumstances of death; ^b^if exercise is not feasible; ^c^re-evaluate if change in family history or new symptoms. Reused from Zeppenfeld *et al.*^86^.

In SADS cases, the subsequent follow-up is dependent on the results of the molecular autopsy of the decedent. Genetic testing of relatives should only be performed if a pathogenic variant has been identified in the decedent.^38,42,86,123,129,130^ If genetic testing of the decedent is negative or has not been performed, it is recommended to refer all first-degree relatives, obligate carriers, and relatives with relevant symptoms for clinical evaluation.^86^ For all relatives of SADS cases, the initial clinical evaluation consists of review of comorbidities, family history including a minimum of three generation pedigree, physical examination, standard and high precordial lead electrocardiogram, exercise test, and echocardiography (Class 1 recommendation in the 2022 European Society of Cardiology (ESC) guidelines on arrhythmia and SCD; *Figure 6*).^42–45,86,126,129–131^ In some cases, it is also relevant to perform ambulatory cardiac rhythm monitoring, signal-averaged electrocardiogram, cardiac magnetic resonance, and provocative testing (Class IIa and IIb recommendation in the 2022 ESC guidelines on arrhythmia and SCD).^42,44,56,86,129,130^ The context of SCD, the family history, and the results of the above-mentioned Class I recommended examinations should inform the potential merits of additional analysis and dictate in which order they are to be performed if at all.

For example, the use of sodium channel blocker provocation testing to diagnose Brugada syndrome is recommended at the level of IIa in both the ESC guidelines and in an expert consensus statement from the Asia Pacific Heart Rhythm Society and the Heart Rhythm Society in post-pubertal family members of SADS cases where the pro-band findings or baseline tests increase the suspicion of Brugada syndrome.^56,86^ The systematic use of pharmacological testing, however, may be considered but only after exclusion of all pathological and clinical diagnoses in the decedents and their relatives due to the potential for inappropriate diagnoses of Brugada syndrome.^132^

Cardiomyopathy may be diagnosed despite a negative autopsy, presumably due to concealed disease in the decedent, and may be confirmed by the molecular autopsy.^109^

In SADS families where no diagnosis have been made after clinical and genetic evaluation, follow-up is not recommended in asymptomatic adults, unless they develop new symptoms or the family history changes, in which case they should be instructed to contact the healthcare system.^86,133,134^ Children of the decedent are recommended to receive follow-up until they reach adulthood.

### Perspectives

Autopsy of unexplained SD in the young is recommended by both the ESC and the American Heart Association. However, even in well-resourced countries, many victims of sudden and unexpected death are not autopsied, likely due to monetary and organizational limitations and lack of awareness.^50^

This leaves the remaining relatives uncertain or unaware of whether other family members are at risk of SD including exclusion of an inherited disease. There are a substantial proportion of non-autopsied SDs in decedents aged 35–50 years, despite evidence of inherited heart disease in this age group.^17^ The authors of this review strongly recommend the use of autopsy in all SDs less than 50 years of age.

There are limited data on the incidence of SCD due to inherited causes in those >50 years, which should be the focus of future research. Dissemination of the knowledge that inherited heart disease often underlies SD all the way up to those aged 50 years is important to increase the uptake of autopsy. Equally important is to inform and influence politicians and other key policy makers in healthcare to increase funding and alter legislation to achieve the goal of autopsying all unexpected SDs in the young and ensure clinical assessment of at-risk family members. Solutions in the health system arena are inevitably local, and precedent and guidelines reinforce the merits, but advocacy including patient and family groups or organizations is often useful in generating influence. The quality of autopsy and heart examination according to standardized protocols with possible referral for second opinion to an expert cardiovascular pathology centre should also be emphasized.

New legislation has recently been implemented in Denmark to increase the autopsy rate among SD cases. Aligned with many other countries, the rate of hospital autopsies in Denmark has been steeply declining. Previously, if there was no suspicion of crime-related death, a forensic autopsy would often not be performed due to lack of capacity and monetary limitations even though the Danish Society of Cardiology recommends autopsy of all SDs < 50 years. The legislative changes now allow for forensic autopsy of SD cases irrespective of whether a crime is suspected, which will increase autopsy rates and consequently enable evaluation of family members of young SCD victims. In the UK, a National Health Service Program has been initiated aimed at systematizing access to post-mortem genetic testing and family screening in inherited Cardiac Condition Centers by creating pathways between Coroners and the health service.^135^

Over the last two decades, there have been large advances in genetic sequencing technologies. This has allowed for cost-efficient tests of large gene panels and sequencing of the entire exome or genome. Recent studies suggest that next-generation sequencing in SCD victims and unexpected cardiac arrest survivors improves diagnostic yield.^107,109,116,117,136–138^ However, broader genetic testing increases the risk of identifying variants of uncertain significance, which is especially challenging to interpret in the setting of SADS, where correlation to the phenotype is impossible in the decedent.^81^ Over-interpretation of clinical or genetic findings of unknown significance might lead to erroneous diagnosis and cause significant harm. The utility of broad panels and whole-genome sequencing, therefore, needs to be carefully tested before full implementation in clinical practice. Further, novel approaches using RNA sequencing alongside DNA sequencing can help discriminate causative variants from variants of uncertain significance and may lead to diagnostic transcriptomic profiles.^139^

## Conclusion

Autopsy rates are declining worldwide, and not all cases of SD are autopsied. A large proportion of all SCD in the young are caused by inherited cardiac disease. Autopsy and post-mortem genetic testing of young SCD cases along with detailed cardiac and genetic investigations of first-degree relatives result in high yield of diagnoses of inherited cardiac disease in the families allowing for risk reduction advice and treatment. A comprehensive autopsy following a standard protocol and often including involvement of a cardiac pathologist is, therefore, essential in all cases of unexpected SD in persons below the age of 50 years. Due to high diagnostic complexity, clinical and genetic evaluation and subsequent management of the families should be carried out by specialized multidisciplinary teams.

## Data Availability

All relevant data are within the manuscript and its supporting information files.

## References

[1] Lynge TH, Risgaard B, Banner J, Nielsen JL, Jespersen T, Stampe NK et al Nationwide burden of sudden cardiac death: a study of 54,028 deaths in Denmark. Heart Rhythm 2021;18:1657–65. S1547-5271(21)00422-7. 33965606 10.1016/j.hrthm.2021.05.005

[2] Fishman GI, Chugh SS, DiMarco JP, Albert CM, Anderson ME, Bonow RO et al Sudden cardiac death prediction and prevention report from a national heart, lung, and blood institute and heart rhythm society workshop. Circulation 2010;122:2335–48. 21147730 10.1161/CIRCULATIONAHA.110.976092 PMC3016224

[3] Zipes DPM, Camm AJM, Borggrefe MM, Buxton AEM, Chaitman BM, Fromer M et al ACC/AHA/ESC 2006 guidelines for management of patients with ventricular arrhythmias and the prevention of sudden cardiac death: a report of the American College of Cardiology/American Heart Association task force and the European Society of Cardiology committee for practice guidelines (writing committee to develop guidelines for management of patients with ventricular arrhythmias and the prevention of sudden cardiac death). Circulation 2006;114:385–484. 10.1161/CIRCULATIONAHA.106.178233 16935995

[4] Hayashi M, Shimizu W, Albert CM. The spectrum of epidemiology underlying sudden cardiac death. Circ Res 2015;116:1887–906. 26044246 10.1161/CIRCRESAHA.116.304521 PMC4929621

[5] Berdowski J, Berg RA, Tijssen JGP, Koster RW. Global incidences of out-of-hospital cardiac arrest and survival rates: systematic review of 67 prospective studies. Resuscitation 2010;81:1479–87. 20828914 10.1016/j.resuscitation.2010.08.006

[6] Priori SG, Aliot E, Blømstrom-Lundqvist C, Bossaert L, Breithardt G, Brugada P et al Task force on sudden cardiac death, European Society of Cardiology. Europace 2002;4:3–18. 11858152 10.1053/eupc.2001.0214

[7] Wong CX, Brown A, Lau DH, Chugh SS, Albert CM, Kalman JM et al Epidemiology of sudden cardiac death: global and regional perspectives. Heart Lung Circ 2019;28:6–14. 30482683 10.1016/j.hlc.2018.08.026

[8] Benjamin EJ, Virani SS, Callaway CW, Chamberlain AM, Chang AR, Cheng S et al Heart disease and stroke statistics-2018 update: a report from the American Heart Association. Circulation 2018;137:e67–492. 29386200 10.1161/CIR.0000000000000558

[9] Marijon E, Narayanan K, Smith K, Barra S, Basso C, Blom MT et al The lancet commission to reduce the global burden of sudden cardiac death: a call for multidisciplinary action. Lancet 2023;402:883–936. 37647926 10.1016/S0140-6736(23)00875-9

[10] Tfelt-Hansen J, Garcia R, Albert C, Merino J, Krahn A, Marijon E et al Risk stratification of sudden cardiac death: a review. Europace 2023;25:euad203. 37622576 10.1093/europace/euad203 PMC10450787

[11] Könemann H, Dagres N, Merino JL, Sticherling C, Zeppenfeld K, Tfelt-Hansen J et al Spotlight on the 2022 ESC guideline management of ventricular arrhythmias and prevention of sudden cardiac death: 10 novel key aspects. Europace 2023;25:euad091. 37102266 10.1093/europace/euad091 PMC10228619

[12] Stecker EC, Reinier K, Marijon E, Narayanan K, Teodorescu C, Uy-Evanado A et al Public health burden of sudden cardiac death in the United States. Circ Arrhythm Electrophysiol 2014;7:212–7. 24610738 10.1161/CIRCEP.113.001034 PMC4041478

[13] Bagnall RD, Weintraub RG, Ingles J, Duflou J, Yeates L, Lam L et al A prospective study of sudden cardiac death among children and young adults. N Engl J Med 2016;374:2441–52. 27332903 10.1056/NEJMoa1510687

[14] Winkel BG, Holst AG, Theilade J, Kristensen IB, Thomsen JL, Ottesen GL et al Nationwide study of sudden cardiac death in persons aged 1–35 years. Eur Heart J 2011;32:983–90. 21131293 10.1093/eurheartj/ehq428

[15] Risgaard B, Winkel BG, Jabbari R, Behr ER, Ingemann-Hansen O, Thomsen JL et al Burden of sudden cardiac death in persons aged 1 to 49 years: nationwide study in Denmark. Circ Arrhythm Electrophysiol 2014;7:205–11. 24604905 10.1161/CIRCEP.113.001421

[16] Deo R, Albert CM. Epidemiology and genetics of sudden cardiac death. Circulation 2012;125:620–37. 22294707 10.1161/CIRCULATIONAHA.111.023838 PMC3399522

[17] Lynge TH, Nielsen JL, Risgaard B, van der Werf C, Winkel BG, Tfelt-Hansen J. Causes of sudden cardiac death according to age and sex in persons aged 1–49 years. Heart Rhythm 2023;20:61–8. 36075534 10.1016/j.hrthm.2022.08.036

[18] Pilmer CM, Porter B, Kirsh JA, Hicks AL, Gledhill N, Jamnik V et al Scope and nature of sudden cardiac death before age 40 in Ontario: a report from the cardiac death advisory committee of the office of the chief coroner. Heart Rhythm 2013;10:517–23. 23232084 10.1016/j.hrthm.2012.12.003

[19] de Vreede-Swagemakers JJM, Gorgels APM, Dubois-Arbouw WI, van Ree JW, Daemen MJAP, Houben LGE et al Out-of-hospital cardiac arrest in the 1990s: a population-based study in the Maastricht area on incidence, characteristics and survival. J Am Coll Cardiol 1997;30:1500–5. 9362408 10.1016/s0735-1097(97)00355-0

[20] Deo R, Norby FL, Katz R, Sotoodehnia N, Adabag S, DeFilippi CR et al Development and validation of a sudden cardiac death prediction model for the general population. Circulation 2016;134:806–16. 27542394 10.1161/CIRCULATIONAHA.116.023042 PMC5021600

[21] Albert CM, Chae CU, Grodstein F, Rose LM, Rexrode KM, Ruskin JN et al Prospective study of sudden cardiac death among women in the United States. Circulation 2003;107:2096–101. 12695299 10.1161/01.CIR.0000065223.21530.11

[22] Priori SG, Blomström-Lundqvist C, Mazzanti A, Blom N, Borggrefe M, Camm J et al 2015 ESC guidelines for the management of patients with ventricular arrhythmias and the prevention of sudden cardiac death: the task force for the management of patients with ventricular arrhythmias and the prevention of sudden cardiac death of the European Society of Cardiology (ESC). endorsed by: Association for European Paediatric and Congenital Cardiology (AEPC). Eur Heart J 2015;36:2793–867. 26320108 10.1093/eurheartj/ehv316

[23] Wissenberg M, Lippert FK, Folke F, Weeke P, Hansen CM, Christensen EF et al Association of national initiatives to improve cardiac arrest management with rates of bystander intervention and patient survival after out-of-hospital cardiac arrest. JAMA 2013;310:1377–84. 24084923 10.1001/jama.2013.278483

[24] Field JM, Hazinski MF, Sayre MR, Chameides L, Schexnayder SM, Hemphill R et al Part 1: executive summary: 2010 American Heart Association guidelines for cardiopulmonary resuscitation and emergency cardiovascular care. Circulation 2010;122:S640–656. 20956217 10.1161/CIRCULATIONAHA.110.970889

[25] Chan PS, McNally B, Tang F, Kellermann A; CARES Surveillance Group. Recent trends in survival from out-of-hospital cardiac arrest in the United States. Circulation 2014;130:1876–82. 25399396 10.1161/CIRCULATIONAHA.114.009711 PMC4276415

[26] Hassager C, Nagao K, Hildick-Smith D. Out-of-hospital cardiac arrest: in-hospital intervention strategies. Lancet 2018;391:989–98. 29536863 10.1016/S0140-6736(18)30315-5

[27] Byrne R, Constant O, Smyth Y, Callagy G, Nash P, Daly K et al Multiple source surveillance incidence and aetiology of out-of-hospital sudden cardiac death in a rural population in the west of Ireland. Eur Heart J 2008;29:1418–23. 18424446 10.1093/eurheartj/ehn155

[28] Chugh SS, Jui J, Gunson K, Stecker EC, John BT, Thompson B et al Current burden of sudden cardiac death: multiple source surveillance versus retrospective death certificate-based review in a large U.S. community. J Am Coll Cardiol 2004;44:1268–75. 15364331 10.1016/j.jacc.2004.06.029

[29] Wang H, Yao Q, Zhu S, Zhang G, Wang Z, Li Z et al The autopsy study of 553 cases of sudden cardiac death in Chinese adults. Heart Vessels 2014;29:486–95. 23836068 10.1007/s00380-013-0388-0

[30] Wilde AAM, Behr ER. Genetic testing for inherited cardiac disease. Nat Rev Cardiol 2013;10:571–83. 23900354 10.1038/nrcardio.2013.108

[31] Sheppard MN, Westaby J, Zullo E, Fernandez BVE, Cox S, Cox A. Sudden arrhythmic death and cardiomyopathy are important causes of sudden cardiac death in the UK: results from a national coronial autopsy database. Histopathology 2023;82:1056–66. 36799099 10.1111/his.14889

[32] Pilmer CM, Kirsh JA, Hildebrandt D, Krahn AD, Gow RM. Sudden cardiac death in children and adolescents between 1 and 19 years of age. Heart Rhythm 2014;11:239–45. 24239636 10.1016/j.hrthm.2013.11.006

[33] Kong MH, Fonarow GC, Peterson ED, Curtis AB, Hernandez AF, Sanders GD et al Systematic review of the incidence of sudden cardiac death in the United States. J Am Coll Cardiol 2011;57:794–801. 21310315 10.1016/j.jacc.2010.09.064 PMC3612019

[34] Kitamura T, Iwami T, Kawamura T, Nitta M, Nagao K, Nonogi H et al Nationwide improvements in survival from out-of-hospital cardiac arrest in Japan. Circulation 2012;126:2834–43. 23035209 10.1161/CIRCULATIONAHA.112.109496

[35] Gillum RF . Geographic variation in sudden coronary death. Am Heart J 1990;119:380–9. 2301226 10.1016/s0002-8703(05)80031-6

[36] Nichol G, Thomas E, Callaway CW, Hedges J, Powell JL, Aufderheide TP et al Regional variation in out-of-hospital cardiac arrest incidence and outcome. JAMA 2008;300:1423–31. 18812533 10.1001/jama.300.12.1423 PMC3187919

[37] Finocchiaro G, Radaelli D, D’Errico S, Papadakis M, Behr ER, Sharma S et al Sudden cardiac death among adolescents in the United Kingdom. J Am Coll Cardiol 2023;81:1007–17. 36922085 10.1016/j.jacc.2023.01.041

[38] Lahrouchi N, Raju H, Lodder EM, Papatheodorou E, Ware JS, Papadakis M et al Utility of post-mortem genetic testing in cases of sudden arrhythmic death syndrome. J Am Coll Cardiol 2017;69:2134–45. 28449774 10.1016/j.jacc.2017.02.046 PMC5405216

[39] Tester DJ, Ackerman MJ. Postmortem long QT syndrome genetic testing for sudden unexplained death in the young. J Am Coll Cardiol 2007;49:240–6. 17222736 10.1016/j.jacc.2006.10.010

[40] Chugh SS, Senashova O, Watts A, Tran PT, Zhou Z, Gong Q et al Postmortem molecular screening in unexplained sudden death. J Am Coll Cardiol 2004;43:1625–9. 15120823 10.1016/j.jacc.2003.11.052

[41] Skinner JR, Crawford J, Smith W, Aitken A, Heaven D, Evans CA et al Prospective, population-based long QT molecular autopsy study of postmortem negative sudden death in 1 to 40 year olds. Heart Rhythm 2011;8:412–9. 21070882 10.1016/j.hrthm.2010.11.016

[42] Behr ER, Dalageorgou C, Christiansen M, Syrris P, Hughes S, Tome Esteban MT et al Sudden arrhythmic death syndrome: familial evaluation identifies inheritable heart disease in the majority of families. Eur Heart J 2008;29:1670–80. 18508782 10.1093/eurheartj/ehn219

[43] McGorrian C, Constant O, Harper N, O’Donnell C, Codd M, Keelan E et al Family-based cardiac screening in relatives of victims of sudden arrhythmic death syndrome. Europace 2013;15:1050–8. 23382499 10.1093/europace/eus408

[44] van der Werf C, Hofman N, Tan HL, van Dessel PF, Alders M, van der Wal AC et al Diagnostic yield in sudden unexplained death and aborted cardiac arrest in the young: the experience of a tertiary referral center in the Netherlands. Heart Rhythm 2010;7:1383–9. 20646679 10.1016/j.hrthm.2010.05.036

[45] Kumar S, Peters S, Thompson T, Morgan N, Maccicoca I, Trainer A et al Familial cardiological and targeted genetic evaluation: low yield in sudden unexplained death and high yield in unexplained cardiac arrest syndromes. Heart Rhythm 2013;10:1653–60. 23973953 10.1016/j.hrthm.2013.08.022

[46] Fellmann F, van El CG, Charron P, Michaud K, Howard HC, Boers SN et al European recommendations integrating genetic testing into multidisciplinary management of sudden cardiac death. Eur J Hum Genet 2019;27:1763–73. 31235869 10.1038/s41431-019-0445-y PMC6870982

[47] Basso C, Aguilera B, Banner J, Cohle S, d’Amati G, de Gouveia RH et al Guidelines for autopsy investigation of sudden cardiac death: 2017 update from the Association for European Cardiovascular Pathology. Virchows Arch 2017;471:691–705. 28889247 10.1007/s00428-017-2221-0 PMC5711979

[48] Sheppard MN, van der Wal AC, Banner J, d’Amati G, De Gaspari M, De Gouveia R et al Genetically determined cardiomyopathies at autopsy: the pivotal role of the pathologist in establishing the diagnosis and guiding family screening. Virchows Arch 2023;482:653–69. 36897369 10.1007/s00428-023-03523-8 PMC10067659

[49] Schwartz PJ, Crotti L. Can a message from the dead save lives? J Am Coll Cardiol 2007;49:247–9. 17222737 10.1016/j.jacc.2006.10.009

[50] Behr ER, Scrocco C, Wilde AAM, Marijon E, Crotti L, Iliodromitis KE et al Investigation on sudden unexpected death in the young (SUDY) in Europe: results of the European Heart Rhythm Association Survey. Europace 2022;24:331–9. 34351417 10.1093/europace/euab176

[51] Wisten A, Forsberg H, Krantz P, Messner T. Sudden cardiac death in 15–35-year olds in Sweden during 1992–99. J Intern Med 2002;252:529–36. 12472914 10.1046/j.1365-2796.2002.01038.x

[52] World Health Organization. Sudden Cardiac Death. World Health Organization Technical Report Series, Report 726. 1985. 3936284

[53] Tseng ZH, Olgin JE, Vittinghoff E, Ursell PC, Kim AS, Sporer K et al Prospective countywide surveillance and autopsy characterization of sudden cardiac death: POST SCD study. Circulation 2018;137:2689–700. 29915095 10.1161/CIRCULATIONAHA.117.033427 PMC6013842

[54] Waldmann V, Bougouin W, Karam N, Dumas F, Sharifzadehgan A, Gandjbakhch E et al Characteristics and clinical assessment of unexplained sudden cardiac arrest in the real-world setting: focus on idiopathic ventricular fibrillation. Eur Heart J 2018;39:1981–7. 29566157 10.1093/eurheartj/ehy098 PMC5982722

[55] Bougouin W, Lamhaut L, Marijon E, Jost D, Dumas F, Deye N et al Characteristics and prognosis of sudden cardiac death in greater Paris: population-based approach from the Paris Sudden Death Expertise Center (Paris-SDEC). Intensive Care Med 2014;40:846–54. 24658912 10.1007/s00134-014-3252-5

[56] Stiles MK, Wilde AAM, Abrams DJ, Ackerman MJ, Albert CM, Behr ER et al 2020 APHRS/HRS expert consensus statement on the investigation of decedents with sudden unexplained death and patients with sudden cardiac arrest, and of their families. Heart Rhythm 2021;37:481–534. 10.1016/j.hrthm.2020.10.010 PMC8194370 33091602

[57] Lynge TH, Nielsen JL, Blanche P, Gislason G, Torp-Pedersen C, Winkel BG et al Decline in incidence of sudden cardiac death in the young: a 10-year nationwide study of 8756 deaths in Denmark. Europace 2019;21:909–17. 30809645 10.1093/europace/euz022

[58] Svane J, Lynge TH, Hansen CJ, Risgaard B, Winkel BG, Tfelt-Hansen J. Witnessed and unwitnessed sudden cardiac death: a nationwide study of persons aged 1–35 years. Europace 2021;23:898–906. 33595080 10.1093/europace/euab017

[59] Risgaard B, Lynge TH, Wissenberg M, Jabbari R, Glinge C, Gislason GH et al Risk factors and causes of sudden noncardiac death: a nationwide cohort study in Denmark. Heart Rhythm 2015;12:968–74. 25614248 10.1016/j.hrthm.2015.01.024

[60] Gullach AJ, Risgaard B, Lynge TH, Jabbari R, Glinge C, Haunsø S et al Sudden death in young persons with uncontrolled asthma - a nationwide cohort study in Denmark. BMC Pulm Med 2015;15. 10.1186/s12890-015-0033-z PMC4404085 25887740

[61] Bohm P, Meyer T, Narayanan K, Schindler M, Weizman O, Beganton F et al Sports-related sudden cardiac arrest in young adults. Europace 2023;25:627–33. 36256586 10.1093/europace/euac172 PMC9935050

[62] Ågesen FN, Risgaard B, Zachariasardóttir S, Jabbari R, Lynge TH, Ingemann-Hansen O et al Sudden unexpected death caused by stroke: a nationwide study among children and young adults in Denmark. Int J Stroke 2017;13:1747493017724625 3. 10.1177/1747493017724625 28762897

[63] Haukilahti MAE, Holmström L, Vähätalo J, Kenttä T, Tikkanen J, Pakanen L et al Sudden cardiac death in women. Circulation 2019;139:1012–21. 30779638 10.1161/CIRCULATIONAHA.118.037702

[64] Winkel BG, Risgaard B, Bjune T, Jabbari R, Lynge TH, Glinge C et al Gender differences in sudden cardiac death in the young-a nationwide study. BMC Cardiovasc Disord 2017;17:19. 28061807 10.1186/s12872-016-0446-5 PMC5219679

[65] Skjelbred T, Rajan D, Svane J, Lynge TH, Tfelt-Hansen J. Sex differences in sudden cardiac death in a nationwide study of 54 028 deaths. Heart 2022;108:1012–8. heartjnl-2021–320300. 35277455 10.1136/heartjnl-2021-320300

[66] Winkel BG, Holst AG, Theilade J, Kristensen IB, Thomsen JL, Hansen SH et al Sudden unexpected death in infancy in Denmark. Scand Cardiovasc J 2011;45:14–20. 21133644 10.3109/14017431.2010.538433

[67] Lynge TH, Jeppesen AG, Winkel BG, Glinge C, Schmidt MR, Søndergaard L et al Nationwide study of sudden cardiac death in people with congenital heart defects aged 0 to 35 years. Circ Arrhythm Electrophysiol 2018;11:e005757. 29858381 10.1161/CIRCEP.117.005757

[68] Tester DJ, Wong LCH, Chanana P, Jaye A, Evans JM, FitzPatrick DR et al Cardiac genetic predisposition in sudden infant death syndrome. J Am Coll Cardiol 2018;71:1217–27. 29544605 10.1016/j.jacc.2018.01.030

[69] Filiano JJ, Kinney HC. A perspective on neuropathologic findings in victims of the sudden infant death syndrome: the triple-risk model. Biol Neonate 1994;65:194–7. 8038282 10.1159/000244052

[70] Baruteau AE, Tester DJ, Kapplinger JD, Ackerman MJ, Behr ER. Sudden infant death syndrome and inherited cardiac conditions. Nat Rev Cardiol 2017;14:715–26. 28880023 10.1038/nrcardio.2017.129

[71] Schwartz PJ, Kotta MC. Sudden infant death syndrome and genetics. J Am Coll Cardiol 2018;71:1228–30. 29544606 10.1016/j.jacc.2018.01.039

[72] Van Norstrand DW, Ackerman MJ. Genomic risk factors in sudden infant death syndrome. Genome Med 2010;2:86. 21122164 10.1186/gm207 PMC3016628

[73] Schwartz PJ . Cardiac sympathetic innervation and the sudden infant death syndrome. A possible pathogenetic link. Am J Med 1976;60:167–72. 175654 10.1016/0002-9343(76)90425-3

[74] Ackerman MJ, Siu BL, Sturner WQ, Tester DJ, Valdivia CR, Makielski JC et al Postmortem molecular analysis of SCN5A defects in sudden infant death syndrome. JAMA 2001;286:2264–9. 11710892 10.1001/jama.286.18.2264

[75] Schwartz PJ, Priori SG, Dumaine R, Napolitano C, Antzelevitch C, Stramba-Badiale M et al A molecular link between the sudden infant death syndrome and the long-QT syndrome. N Engl J Med 2000;343:262–7. 10911008 10.1056/NEJM200007273430405

[76] Arnestad M, Crotti L, Rognum TO, Insolia R, Pedrazzini M, Ferrandi C et al Prevalence of long-QT syndrome gene variants in sudden infant death syndrome. Circulation 2007;115:361–7. 17210839 10.1161/CIRCULATIONAHA.106.658021

[77] Kotta M, Torchio M, Bayliss P, Cohen MC, Quarrell O, Wheeldon N et al Cardiac genetic investigation of sudden infant and early childhood death: a study from victims to families. J Am Heart Assoc 2023;12:e029100. 37589201 10.1161/JAHA.122.029100 PMC10547337

[78] Lynge TH, Risgaard B, Jabbari R, Glinge C, Bundgaard H, Maron B et al Cardiac symptoms before sudden cardiac death caused by hypertrophic cardiomyopathy: a nationwide study among the young in Denmark. Europace 2016;18:1801–8. 26823388 10.1093/europace/euv403

[79] Agbaedeng TA, Roberts KA, Colley L, Noubiap JJ, Oxborough D. Incidence and predictors of sudden cardiac death in arrhythmogenic right ventricular cardiomyopathy: a pooled analysis. Europace 2022;24:1665–74. 35298614 10.1093/europace/euac014 PMC9559905

[80] Gasperetti A, James CA, Carrick RT, Protonotarios A, Te Riele ASJM, Cadrin-Tourigny J et al Arrhythmic risk stratification in arrhythmogenic right ventricular cardiomyopathy. Europace 2023;25:euad312. 37935403 10.1093/europace/euad312 PMC10674106

[81] Crotti L, Brugada P, Calkins H, Chevalier P, Conte G, Finocchiaro G et al From gene-discovery to gene-tailored clinical management: 25 years of research in channelopathies and cardiomyopathies. Europace 2023;25:euad180. 37622577 10.1093/europace/euad180 PMC10450790

[82] Paul T, Krause U, Sanatani S, Etheridge SP. Advancing the science of management of arrhythmic disease in children and adult congenital heart disease patients within the last 25 years. Europace 2023;25:euad155. 37622573 10.1093/europace/euad155 PMC10450816

[83] Chappex N, Schlaepfer J, Fellmann F, Bhuiyan ZA, Wilhelm M, Michaud K. Sudden cardiac death among general population and sport related population in forensic experience. J Forensic Leg Med 2015;35:62–8. 26344462 10.1016/j.jflm.2015.07.004

[84] Myerburg RJ, Junttila MJ. Sudden cardiac death caused by coronary heart disease. Circulation 2012;125:1043–52. 22371442 10.1161/CIRCULATIONAHA.111.023846

[85] Bowker TJ, Wood DA, Davies MJ, Sheppard MN, Cary NRB, Burton JDK et al Sudden, unexpected cardiac or unexplained death in England: a national survey. QJM 2003;96:269–79. 12651971 10.1093/qjmed/hcg038

[86] Zeppenfeld K, Tfelt-Hansen J, de Riva M, Winkel BG, Behr ER, Blom NA et al 2022 ESC guidelines for the management of patients with ventricular arrhythmias and the prevention of sudden cardiac death. Eur Heart J 2022;43:3997–4126. 36017572 10.1093/eurheartj/ehac262

[87] Zhao D, Post WS, Blasco-Colmenares E, Cheng A, Zhang Y, Deo R et al Racial differences in sudden cardiac death. Circulation 2019;139:1688–97. 30712378 10.1161/CIRCULATIONAHA.118.036553 PMC6443438

[88] Garcia R, Rajan D, Warming PE, Svane J, Vissing C, Weeke P et al Ethnic disparities in out-of-hospital cardiac arrest: a population-based cohort study among adult Danish immigrants. Lancet Reg Health Eur 2022;22:100477. 35957808 10.1016/j.lanepe.2022.100477 PMC9361311

[89] Maruyama M, Ohira T, Imano H, Kitamura A, Kiyama M, Okada T et al Trends in sudden cardiac death and its risk factors in Japan from 1981 to 2005: the circulatory risk in communities study (CIRCS). BMJ Open 2012;2:e000573. 10.1136/bmjopen-2011-000573 PMC3312077 22446988

[90] Hua W, Zhang LF, Wu YF, Liu XQ, Guo DS, Zhou HL et al Incidence of sudden cardiac death in China: analysis of 4 regional populations. J Am Coll Cardiol 2009;54:1110–8. 19744622 10.1016/j.jacc.2009.06.016

[91] Shojania KG, Burton EC. The vanishing nonforensic autopsy. N Engl J Med 2008;358:873–5. 18305264 10.1056/NEJMp0707996

[92] Sington JD, Cottrell BJ. Analysis of the sensitivity of death certificates in 440 hospital deaths: a comparison with necropsy findings. J Clin Pathol 2002;55:499–502. 12101193 10.1136/jcp.55.7.499 PMC1769693

[93] Kircher T, Nelson J, Burdo H. The autopsy as a measure of accuracy of the death certificate. N Engl J Med 1985;313:1263–9. 4058507 10.1056/NEJM198511143132005

[94] Basso C, Stone JR. Autopsy in the era of advanced cardiovascular imaging. Eur Heart J 2022;43:2461–8. 35514073 10.1093/eurheartj/ehac220 PMC9336584

[95] Larsen ST, Lynnerup N. Medico-legal autopsies in Denmark. Dan Med Bull 2011;58:A4247. 21371404

[96] van der Werf C, Hendrix A, Birnie E, Bots ML, Vink A, Bardai A et al Improving usual care after sudden death in the young with focus on inherited cardiac diseases (the CAREFUL study): a community-based intervention study. Europace 2016;18:592–601. 25833117 10.1093/europace/euv059

[97] Svane J, Nielsen JL, Stampe NK, Feldt-Rasmussen B, Garcia R, Risgaard B et al Nationwide study of mortality and sudden cardiac death in young persons diagnosed with chronic kidney disease. Europace 2022;24:euac032. 10.1093/europace/euac032 35373838

[98] Kløvgaard M, Lynge TH, Tsiropoulos I, Uldall PV, Banner J, Winkel BG et al Sudden unexpected death in epilepsy in persons younger than 50 years: a retrospective nationwide cohort study in Denmark. Epilepsia 2021;62:2405–241. 34418071 10.1111/epi.17037

[99] Lynge TH, Svane J, Pedersen-Bjergaard U, Gislason G, Torp-Pedersen C, Banner J et al Sudden cardiac death among persons with diabetes aged 1–49 years: a 10-year nationwide study of 14 294 deaths in Denmark. Eur Heart J 2020;41:2699–706. 31848583 10.1093/eurheartj/ehz891 PMC7377578

[100] Banner J, Basso C, Tolkien Z, Kholova I, Michaud K, Gallagher PJ. Autopsy examination in sudden cardiac death: a current perspective on behalf of the Association for European Cardiovascular Pathology. Virchows Arch 2021;478:687–93. 33111163 10.1007/s00428-020-02949-8 PMC7990811

[101] Lynge TH, Nielsen TS, Gregers Winkel B, Tfelt-Hansen J, Banner J. Sudden cardiac death caused by myocarditis in persons aged 1–49 years: a nationwide study of 14 294 deaths in Denmark. Forensic Sci Res 2019;4:247–56. 31489390 10.1080/20961790.2019.1595352 PMC6713107

[102] Kelly KL, Lin PT, Basso C, Bois M, Buja LM, Cohle SD et al Sudden cardiac death in the young: a consensus statement on recommended practices for cardiac examination by pathologists from the Society for Cardiovascular Pathology. Cardiovasc Pathol 2023;63:107497. 36375720 10.1016/j.carpath.2022.107497

[103] Sheppard MN, Kim Suvarna S. Guidelines on autopsy practice: sudden death with likely cardiac pathology. R Coll Pathol 2022;G145:1–17.

[104] de Noronha SV, Behr ER, Papadakis M, Ohta-Ogo K, Banya W, Wells J et al The importance of specialist cardiac histopathological examination in the investigation of young sudden cardiac deaths. Europace 2014;16:899–907. 24148315 10.1093/europace/eut329

[105] Sheppard MN . The autopsy is still valuable: national registries and promoting autopsy after sudden cardiac death. Heart Rhythm 2024;21:684–5. S1547–5271(24)00091–2. 38280623 10.1016/j.hrthm.2024.01.037

[106] Wilde AAM, Semsarian C, Márquez MF, Shamloo AS, Ackerman MJ, Ashley EA et al European Heart Rhythm Association (EHRA)/Heart Rhythm Society (HRS)/Asia Pacific Heart Rhythm Society (APHRS)/Latin American Heart Rhythm Society (LAHRS) expert consensus statement on the state of genetic testing for cardiac diseases. Europace 2022;24:1307–67. 35373836 10.1093/europace/euac030 PMC9435643

[107] Grondin S, Davies B, Cadrin-Tourigny J, Steinberg C, Cheung CC, Jorda P et al Importance of genetic testing in unexplained cardiac arrest. Eur Heart J 2022;43:3071–81. 35352813 10.1093/eurheartj/ehac145 PMC9392649

[108] Bjune T, Risgaard B, Kruckow L, Glinge C, Ingemann-Hansen O, Leth PM et al Post-mortem toxicology in young sudden cardiac death victims: a nationwide cohort study. Europace 2018;20:614–21. 28339816 10.1093/europace/euw435

[109] Isbister JC, Nowak N, Yeates L, Singer ES, Sy RW, Ingles J et al Concealed cardiomyopathy in autopsy-inconclusive cases of sudden cardiac death and implications for families. J Am Coll Cardiol 2022;80:2057–68. 36423990 10.1016/j.jacc.2022.09.029

[110] Goldman AM, Behr ER, Semsarian C, Bagnall RD, Sisodiya S, Cooper PN. Sudden unexpected death in epilepsy genetics: molecular diagnostics and prevention. Epilepsia 2016;57:17–25. 26749013 10.1111/epi.13232 PMC5034873

[111] Chahal CAA, Tester DJ, Fayyaz AU, Jaliparthy K, Khan NA, Lu D et al Confirmation of cause of death via comprehensive autopsy and whole exome molecular sequencing in people with epilepsy and sudden unexpected death. J Am Heart Assoc 2021;10:e021170. 34816733 10.1161/JAHA.121.021170 PMC9075361

[112] Al-Khatib SM, Stevenson WG, Ackerman MJ, Gillis AM, Bryant WJ, Hlatky MA et al 2017 AHA/ACC/HRS guideline for management of patients with ventricular arrhythmias and the prevention of sudden cardiac death: executive summary: a report of the American College of Cardiology/American Heart Association task force on clinical practice guidelines and the Heart Rhythm Society. Heart Rhythm 2017;138:e210–71. 10.1161/CIR.0000000000000548 29084733

[113] Lahrouchi N, Raju H, Lodder EM, Papatheodorou S, Miles C, Ware JS et al The yield of postmortem genetic testing in sudden death cases with structural findings at autopsy. Eur J Hum Genet 2020;28:17–22. 31534214 10.1038/s41431-019-0500-8 PMC6906523

[114] Tester DJ, Medeiros-Domingo A, Will ML, Haglund CM, Ackerman MJ. Cardiac channel molecular autopsy: insights from 173 consecutive cases of autopsy-negative sudden unexplained death referred for postmortem genetic testing. Mayo Clin Proc 2012;87:524–39. 22677073 10.1016/j.mayocp.2012.02.017 PMC3498431

[115] Raju H, Ware JS, Skinner JR, Hedley PL, Arno G, Love DR et al Next-generation sequencing using microfluidic PCR enrichment for molecular autopsy. BMC Cardiovasc Disord 2019;19:174. 31337358 10.1186/s12872-019-1154-8 PMC6651896

[116] Lahrouchi N, Behr ER, Bezzina CR. Next-generation sequencing in post-mortem genetic testing of young sudden cardiac death cases. Front Cardiovasc Med 2016;3:13. 27303672 10.3389/fcvm.2016.00013 PMC4885007

[117] Semsarian C, Ingles J, Wilde AAM. Sudden cardiac death in the young: the molecular autopsy and a practical approach to surviving relatives. Eur Heart J 2015;36:1290–6. 25765769 10.1093/eurheartj/ehv063

[118] Pannone L, Bisignani A, Osei R, Gauthey A, Sorgente A, Vergara P et al Genetic testing in children with Brugada syndrome: results from a large prospective registry. Europace 2023;25:euad079. 37061847 10.1093/europace/euad079 PMC10227762

[119] Anderson JH, Tester DJ, Will ML, Ackerman MJ. Whole-exome molecular autopsy after exertion-related sudden unexplained death in the young. Circ Cardiovasc Genet 2016;9:259–65. 27114410 10.1161/CIRCGENETICS.115.001370

[120] Crotti L, Spazzolini C, Tester DJ, Ghidoni A, Baruteau AE, Beckmann BM et al Calmodulin mutations and life-threatening cardiac arrhythmias: insights from the International Calmodulinopathy Registry. Eur Heart J 2019;40:2964–75. 31170290 10.1093/eurheartj/ehz311 PMC6748747

[121] Crotti L, Spazzolini C, Nyegaard M, Overgaard MT, Kotta MC, Dagradi F et al Clinical presentation of calmodulin mutations: the International Calmodulinopathy Registry. Eur Heart J 2023;44:3357–70. 37528649 10.1093/eurheartj/ehad418 PMC10499544

[122] Coelho-Lima J, Westaby J, Sheppard MN. Cardiac arrest with successful cardiopulmonary resuscitation and survival induce histologic changes that correlate with survival time and lead to misdiagnosis in sudden arrhythmic death syndrome. Resuscitation 2022;175:6–12. 35405310 10.1016/j.resuscitation.2022.04.002

[123] Papadakis M, Raju H, Behr ER, De Noronha SV, Spath N, Kouloubinis A et al Sudden cardiac death with autopsy findings of uncertain significance: potential for erroneous interpretation. Circ Arrhythm Electrophysiol 2013;6:588–96. 23671135 10.1161/CIRCEP.113.000111

[124] Yeates L, Hunt L, Saleh M, Semsarian C, Ingles J. Poor psychological wellbeing particularly in mothers following sudden cardiac death in the young. Eur J Cardiovasc Nurs 2013;12:484–91. 23568895 10.1177/1474515113485510

[125] Dalgaard CV, Hansen BL, Jacobsen EM, Kjerrumgaard A, Tfelt-Hansen J, Weeke PE et al Sudden unexplained death versus nonautopsied possible sudden cardiac death: findings in relatives. J Cardiovasc Electrophysiol 2022;33:254–61. 34918422 10.1111/jce.15333

[126] Behr E, Wood DA, Wright M, Syrris P, Sheppard MN, Casey A et al Cardiological assessment of first-degree relatives in sudden arrhythmic death syndrome. Lancet 2003;362:1457–9. 14602442 10.1016/s0140-6736(03)14692-2

[127] Hansen BL, Jacobsen EM, Kjerrumgaard A, Tfelt-Hansen J, Winkel BG, Bundgaard H et al Diagnostic yield in victims of sudden cardiac death and their relatives. Europace 2020;22:964–71. 32307520 10.1093/europace/euaa056

[128] Aktaa S, Tzeis S, Gale CP, Ackerman MJ, Arbelo E, Behr ER et al European Society of Cardiology quality indicators for the management of patients with ventricular arrhythmias and the prevention of sudden cardiac death. Europace 2023;25:199–210. 36753478 10.1093/europace/euac114 PMC10103575

[129] Tan HL, Hofman N, van Langen IM, van der Wal AC, Wilde AAM. Sudden unexplained death: heritability and diagnostic yield of cardiological and genetic examination in surviving relatives. Circulation 2005;112:207–13. 15998675 10.1161/CIRCULATIONAHA.104.522581

[130] Papadakis M, Papatheodorou E, Mellor G, Raju H, Bastiaenen R, Wijeyeratne Y et al The diagnostic yield of Brugada syndrome after sudden death with normal autopsy. J Am Coll Cardiol 2018;71:1204–14. 29544603 10.1016/j.jacc.2018.01.031

[131] Mellor G, Raju H, de Noronha SV, Papadakis M, Sharma S, Behr ER et al Clinical characteristics and circumstances of death in the sudden arrhythmic death syndrome. Circ Arrhythm Electrophysiol 2014;7:1078–83. 25262685 10.1161/CIRCEP.114.001854

[132] Tadros R, Nannenberg EA, Lieve KV, Škorić-Milosavljević D, Lahrouchi N, Lekanne Deprez RH et al Yield and pitfalls of ajmaline testing in the evaluation of unexplained cardiac arrest and sudden unexplained death: single-center experience with 482 families. JACC Clin Electrophysiol 2017;3:1400–8. 29759671 10.1016/j.jacep.2017.04.005

[133] Wong LCH, Roses-Noguer F, Till JA, Behr ER. Cardiac evaluation of pediatric relatives in sudden arrhythmic death syndrome: a 2-center experience. Circ Arrhythm Electrophysiol 2014;7:800–6. 25194972 10.1161/CIRCEP.114.001818

[134] van der Werf C, Stiekema L, Tan HL, Hofman N, Alders M, van der Wal AC et al Low rate of cardiac events in first-degree relatives of diagnosis-negative young sudden unexplained death syndrome victims during follow-up. Heart Rhythm 2014;11:1728–32. 24882506 10.1016/j.hrthm.2014.05.028

[135] Sudden Unexpected Death—Central and South Genomics Service. [Last accessed 24th of February]. Available at: https://centralsouthgenomics.nhs.uk/transformation-projects-patients/sudden-unexpected-death/

[136] Bagnall RD, Das KJ, Duflou J, Semsarian C. Exome analysis-based molecular autopsy in cases of sudden unexplained death in the young. Heart Rhythm 2014;11:655–62. 24440382 10.1016/j.hrthm.2014.01.017

[137] Loporcaro CG, Tester DJ, Maleszewski JJ, Kruisselbrink T, Ackerman MJ. Confirmation of cause and manner of death via a comprehensive cardiac autopsy including whole exome next-generation sequencing. Arch Pathol Lab Med 2014;138:1083–9. 24298987 10.5858/arpa.2013-0479-SA

[138] Isbister JC, Nowak N, Butters A, Yeates L, Gray B, Sy RW et al Concealed cardiomyopathy” as a cause of previously unexplained sudden cardiac arrest. Int J Cardiol 2021;324:96–101. 32931854 10.1016/j.ijcard.2020.09.031

[139] Bueno-Beti C, Johnson DC, Miles C, Westaby J, Sheppard MN, Behr ER et al Potential diagnostic role for a combined postmortem DNA and RNA sequencing for Brugada syndrome. Circ Genomic Precis Med 2023;16:1–8. 10.1161/CIRCGEN.122.004251 PMC10729895 37795608

